# Textile-Based Wearable Sensor for Skin Hydration Monitoring

**DOI:** 10.3390/s22186985

**Published:** 2022-09-15

**Authors:** Minju Jang, Ho-Dong Kim, Hyung-Jun Koo, Ju-Hee So

**Affiliations:** 1 Material & Component Convergence R&D Department, Korea Institute of Industrial Technology, 143 Hanggaul-ro, Sangnok-gu, Ansan 15588, Korea; 2 Department of Fiber System Engineering, Dankook University, 152 Jukjeon-ro, Suji-gu, Yongin 16890, Korea; 3 Department of Chemical & Biomolecular Engineering, Seoul National University of Science & Technology, 232 Gongneung-ro, Nowon-gu, Seoul 01811, Korea; 4 Department of New and Renewable Energy Convergence, Seoul National University of Science & Technology, 232 Gongneung-ro, Nowon-gu, Seoul 01811, Korea

**Keywords:** wearable sensor, textile sensor, skin hydration, skin impedance, skin monitoring

## Abstract

This research describes a wearable skin hydration sensor based on cotton textile to determine the state of hydration within the skin via impedance analysis. The sensor structure comprises a textile substrate, thermoplastic over-layer, conductive patterns, and encapsulant, designed for stable and reliable monitoring of the skin’s impedance change in relation to hydration level. The porcine skin with different hydration levels was prepared as a model system of the skin, and the textile-based sensor carefully investigated the porcine skin samples’ impedance characteristics. The impedance study reveals that (1) the total impedance of skin decreases as its hydration level increases, and (2) the impedance of the stratum corneum and epidermis layers are more dominantly affected by the hydration level of the skin than the dermis layer. Even after repetitive bending cycles, the impedance data of skin measured by the sensor exhibit a reliable dependence on the skin hydration level, which validates the flexibility and durability of the sensor. Finally, it is shown that the textile-based skin hydration sensor can detect various body parts’ different hydration levels of human skin while maintaining a stable conformal contact with the skin. The resulting data are well-matched with the readings from a commercial skin hydration sensor.

## 1. Introduction

Human and other mammals’ skin regulates body temperature, prevents excessive water loss, and protects the underlying internal organs against harmful stimuli such as pathogens. When skin is damaged due to scarring, inflammation, and diseases such as atopic dermatitis, psoriasis, and xerodermia, it shows functional abnormalities in regulating transepidermal water loss (TEWL) [1,2]. Therefore, the skin hydration level is one of the indicators to diagnose health conditions of skin [3]. Furthermore, the skin’s hydration level affects the skin’s elasticity and resiliency, thereby performing both aesthetic and social functions. Accordingly, academic and commercial interest in monitoring skin hydration levels keeps increasing, and the market is also expanding [4,5,6].

The measurement of electrical characteristics of the skin, such as impedance, offers a simple and noninvasive way to precisely monitor the skin hydration level. As the change in skin hydration level causes the variation in electrical characteristics of the skin, electrodermal activity has been used to monitor physiological or sympathetic conditions that can affect the skin hydration level. There are three different ways to monitor skin hydration level depending on the electric signals to measure [7]. First, the simplest way is to monitor skin resistance or conductance, commonly known as galvanic skin response. Under the direct current (DC) or alternating current (AC) at a fixed frequency, the conductance between two electrodes is measured. The skin conductance sensor is known to have high accuracy in measuring hydration levels for the outermost layer of skin, such as the stratum corneum (SC). However, these measurements are valid for intact skin and have relatively low reliability for diagnosing pathologic skin conditions compared to other measurement methods [8]. Second, the capacitance of the skin can also be used to evaluate how much the skin is hydrated as the relative permittivity of the skin changes with its hydration level. The capacitance-based measurement is called corneometry, named after the first commercial instrument, Corneometer [9]. In healthy skin, both the conductance and capacitance values show reliable dependence on skin hydration level. However, the dependence becomes less reliable for the skin with very low water content, such as palmoplantar skin and skin lesions [8]. The measurement of total impedance of skin provides more comprehensive information on the skin’s hydration level compared to the separate measurement of conductance and capacitance. The impedance analysis enables us to derive the conductance of individual layers constituting the skin, i.e., the SC, epidermis, and dermis, and capacitance at the interfaces between the layers [10,11,12]. Several studies have measured skin impedance with wearable sensors due to these advantages [13,14]. For example, S. Yao et al. measured the impedance of skin using a film-type wearable sensor by applying AC with a varying frequency from 10 to 100 kHz. The magnitude of total impedance decreased with increasing hydration level of the skin [5].

It is important to form a conformal contact between the device and the skin for the accurate and reliable measurement of electric signals of skin. Numerous studies have been conducted to incorporate the patch-type skin hydration sensors as novel technologies for continuous monitoring of physiological data since skin-interfaced wearable sensors first became available. The patch-type wearable skin hydration sensors can reduce the pressure applied to the skin during the measurement and allow continuous monitoring for a longer time than the currently commercially available handheld bar-type skin hydration sensors. Furthermore, if the wearable skin hydration sensors are made of flexible materials such as polymer thin films, they can cover a lot of skin while yet maintaining conformal contact with curved body portions. For example, T. Q. Trung et al. reported a conformal wearable sensor based on polydimethylsiloxane (PDMS) elastomer for continuous skin hydration monitoring [8]. The composites of conductive reduced graphene oxides and polyurethane detect the change in skin resistance depending on its hydration level. Among the flexible sensors for skin hydration monitoring, a few conducted impedance spectroscopies. For instance, X. Huang et al. reported an ultrathin polyimide-based skin hydration sensor referred to as epidermal electronics. The resulting sensor was extremely thin and successfully attached to the skin via van der Waals forces, allowing the stable impedance spectra of the skin to be obtained [15].

The flexibility and wearability of a skin sensor could be increased by using textile as its substrate. In this study, we fabricated a wearable skin hydration sensor based on a textile substrate and conducted an impedance study to determine the hydration levels inside the skin. The textile-based sensor with high flexibility could obtain a stable signal due to the conformal contact with the skin of the curved body parts such as the face, elbows, and heels. A woven cotton textile was chosen as a substrate of the skin hydration sensor as it is hypoallergenic, highly permeable to air, and thus widely used in medical and cosmetic applications [16,17]. Although polymer films are more stable for lithographic processes, we prove that a textile-based skin hydration sensor can be fabricated by simple processes without expensive instruments. A silver paste was printed on the textile substrate by the screen-printing method to form the electrode patterns for impedance measurement. Afterward, additional encapsulation material and a conductive adhesive were applied to enhance the stability and reliability of the textile-based sensor. The moisture content of the pieces of porcine skin was varied by drying or soaking them in a saline solution to investigate the effect of skin hydration level on its impedance. In-depth impedance analysis of the skin samples with different hydration levels was conducted using the textile-based sensor while applying an AC field with varied frequencies. Various impedance parameters were derived, such as the magnitude of total impedance, phase shift, and the resistance and capacitance of the constituting layers of the skin (e.g., SC, epidermis, and dermis) based on the simplified equivalent circuit for the skin. The impedance parameters’ dependence on the skin’s hydration level is discussed. The impedance values measured by the sensor were compared before and after repetitive bending cycles. Finally, we estimated the hydration level of human skin of different body parts from the impedance results measured by the textile-based skin hydration sensor. The accuracy of experimental results by the textile hydration sensor was verified by comparing them with the readings from the commercially available skin moisture meter.

## 2. Materials and Methods

### 2.1. Fabrication of Sensor

**Figure 1:**
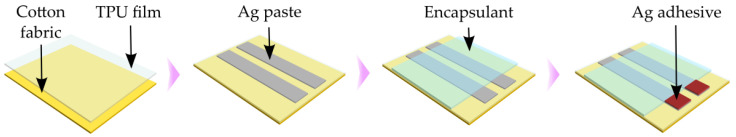
Fabrication steps of a textile-based skin hydration sensor.

Figure 1 shows the fabrication process for the textile-based skin hydration sensor. The cotton textile substrate (plain weave, warp 80′S, and weft 100′S) was purchased from Taihan Textile Co., Ltd., Republic of Korea, and cut into a rectangular piece of 10 cm × 10 cm. First, the thermoplastic polyurethane (TPU) film (Sealon, Seoul, Korea, 3060L) with 50 µm in thickness was hot-pressed on the cotton fabric at 110 °C. After aligning the mask for screen-printing on the textile substrate, the Ag paste (Henkel, Düsseldorf, Germany, LOCTITE EDAG 479SS E&C) and encapsulant (Dycotec, Calne, England, DM-ENC-2500) were sequentially printed on each designated area. For each screen-printing step, the Ag paste and encapsulant films were dried at 120 °C for 10 min. Ag-based adhesive (Creative materials, Massachusetts, USA, 122-44) was applied to the ends of the Ag paste electrodes and dried at 60 °C for 10 min. Afterward, it was stored at room temperature for at least one week to obtain the best adhesiveness and sensing performance.

### 2.2. Determination of Moisture Content in Porcine Skin Using Thermogravimetric Analysis and Histological Study

The back skin of porcine having a thickness of 300 μm (Apures, Pyeongtaek, Korea, Micropic FCM) in a frozen state was purchased and cut into rectangular pieces of 2 cm × 2 cm for the experiments. The porcine skin was either thawed only as a control, which is performed for 30 min at 4 °C, or dehydrated at 25 °C for 30 min after thawing, or hydrated by immersing it in a saline solution for 30 min, 1 h, or 4 h to control the hydration level of the skin. To obtain a quantitative value for skin moisture content, the weight change in porcine skin was measured using thermogravimetric analysis (TGA) (Scinco, Seoul, Korea, TGA N-1000). The temperature was increased from 25 to 200 °C at a rate of 20 °C/min. The measurement was repeated three times each. The pieces of porcine skin with different hydration levels were embedded in paraffin wax, then cut into sections with 4 μm thickness to visualize the change in each layer of skin depending on degree of hydration. The sections were stained with hematoxylin and eosin and scanned using a slide scanner (Zeiss, Oberkochen, Germany, Axio Scan.Z1).

### 2.3. Electrochemical Impedance Spectroscopy (EIS) Measurement of the Textile-Based Skin Hydration Sensor

The skin’s impedance characteristics were investigated using a textile-based sensor with an impedance analyzer (WonATech, Seoul, Korea, ZIVE MP1). After forming a conformal contact between the sensing parts of the sensor and the surface of porcine skin, AC was used with 1 V amplitude without DC offset and a frequency ranging from $10^{0}$ to $10^{5}$ Hz.

### 2.4. Cyclic Bending Test of the Textile-Based Skin Hydration Sensor

A repetitive bending test was conducted using stretching and bending equipment (SNM, Gumi, Korea, Bending & Stretchable Machine System) to examine the flexibility and durability of the textile-based sensor against physical deformation. The sensor was clamped at the ends with two jigs of the equipment. One jig was stationary, and the other jig repeatedly moved back and forth at a rate of 20 mm/s by changing the distance between them from 50 to 6 mm. The sensor was bent 100, 500 or 1000 times with a 3 mm radius of curvature. The resistance of the electrodes and the impedance of the skin before and after the bending test were measured and compared.

### 2.5. Skin Hydration Sensing on Human Skin

The textile-based skin hydration sensor measured the impedance of human skin of various body parts, including the cheek, elbow, hand, and heel. Additionally, the impedance of the back of the hand was measured as a function of the time elapsed after washing with soap. Except for the hands washed with soap, the skin’s surface was sterilized with an alcohol swap before each measurement, and then the sensor was placed on the skin and fixed with medical tape (3M, Minnesota, USA, 1533-1PP). The impedance measured with the textile-based sensor was compared with the data from a commercially available skin hydration sensor (KAKUSAN, Guangdong, China, SK-IV).

## 3. Results and Discussion

### 3.1. Structure of the Textile-Based Skin Hydration Sensor

For monitoring the hydration level in the skin, the configuration of the sensor electrodes should be carefully designed as it determines the depth of electric field penetrating the skin and thus the resulting performance of the sensor. Typically, the thickness of the skin is reported to be 10–30 μm for SC, 31–637 μm for the epidermis, and 469–1942 μm for the dermis [18,19,20]. We designed the electrodes of the sensor as two parallel line patterns with a length of 60 mm, a width of 5 mm, and a gap between them of 1 mm. The penetration depth increases with the width of the electrode [21,22,23]. When the electrode width is too small, the depth cannot cover all the layers of skin during the measurements, and the fabrication processes become complicated. When the electrode width is too large, the depth of the electric field overshoots the thickness of skin that needs to be analyzed, and the sensor size becomes unnecessarily large. As the penetration depth is comparable to the width of an electrode according to the calculation of the previous study (i.e., $T=\frac{a}{2}\sqrt{{\left(1+\frac{2w}{a}\right)}^{2}-1}$, where *T* is the penetration depth, *a* is the gap between the two electrodes, and *w* is the width of the electrode), we chose the width of the electrodes to be 5 mm, which lies in the same order of magnitude as the skin thickness. For the gap of the electrode, it is reported that the penetration depth of the electric field decreases with the gap, and a gap that is less than 1 mm wide is effective in monitoring the electrical characteristics of the skin [24]. The penetration depth of the electric field of the sensor with the gap between electrodes of 1 mm and the width of 5 mm is about 5.48 mm [25], which is large enough to cover the whole thickness of the skin. Thus, we chose the gap to be 1 mm. It should be noted that the frequency of the electric field also affects the penetration depth—a higher frequency creates a shallower electric field in the skin [26]. We adopted the frequency range of the AC field from 1 Hz to 100 kHz, which is previously reported to cover the entire thickness of skin [5].

**Figure 2:**
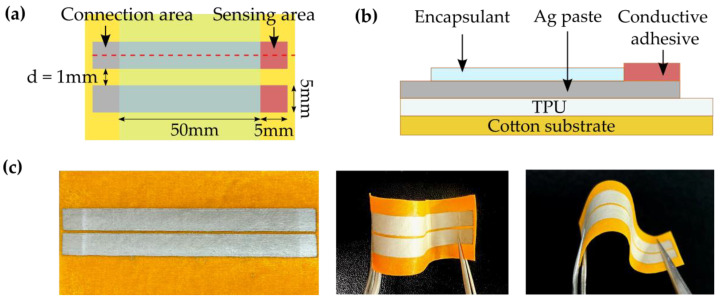
(**a**,**b**) Schematics on the top (**a**) and side (**b**) view of the textile-based skin hydration sensor. (**c**) Digital images of the textile-based skin hydration sensor.

Figure 2 shows the top and side view of the fabricated textile sensor and its digital images. Cotton was chosen as the textile substrate for the sensor. It is widely used for medical applications and cosmetic products because of its excellent wearing comfort, hypoallergenicity, and air permeability. Since the woven cotton textile inherently possesses a hairy, porous, and rough structure, the TPU over-layer film was laminated on the cotton substrate, which provides a smooth flat surface for a more stable formation of electrodes with higher uniformity and flexibility. As designed, the Ag paste, with a sheet resistivity of less than 0.02 Ω/sq, was printed into two parallel lines on the cotton substrate to form electrodes. The ends of the electrodes (the right side of the electrodes in the schematic shown in Figure 2a) are the sensing areas where the electrodes form direct contact with the skin. The areas are covered with an additional conductive adhesive to form a conformal contact with the skin. The adhesive has a resistivity of $1\times 10^{-4}$ Ω∙cm in the Z direction and a peel strength of 1.5–1.8 kN/m. The other ends of each electrode (the left side of the electrodes in the schematic in Figure 2a) are connected to an external impedance analyzer. The surface of the electrodes, excluding the sensing and connection areas, is covered with an insulating encapsulant based on urethane resin. The encapsulant separates the connection area from the sensing area for more accurate and stable measurements of electric signals. The encapsulant also improves the durability of the sensor while maintaining its flexibility.

### 3.2. Dependence of Moisture Content of Porcine Skin on Storage Conditions

**Figure 3:**
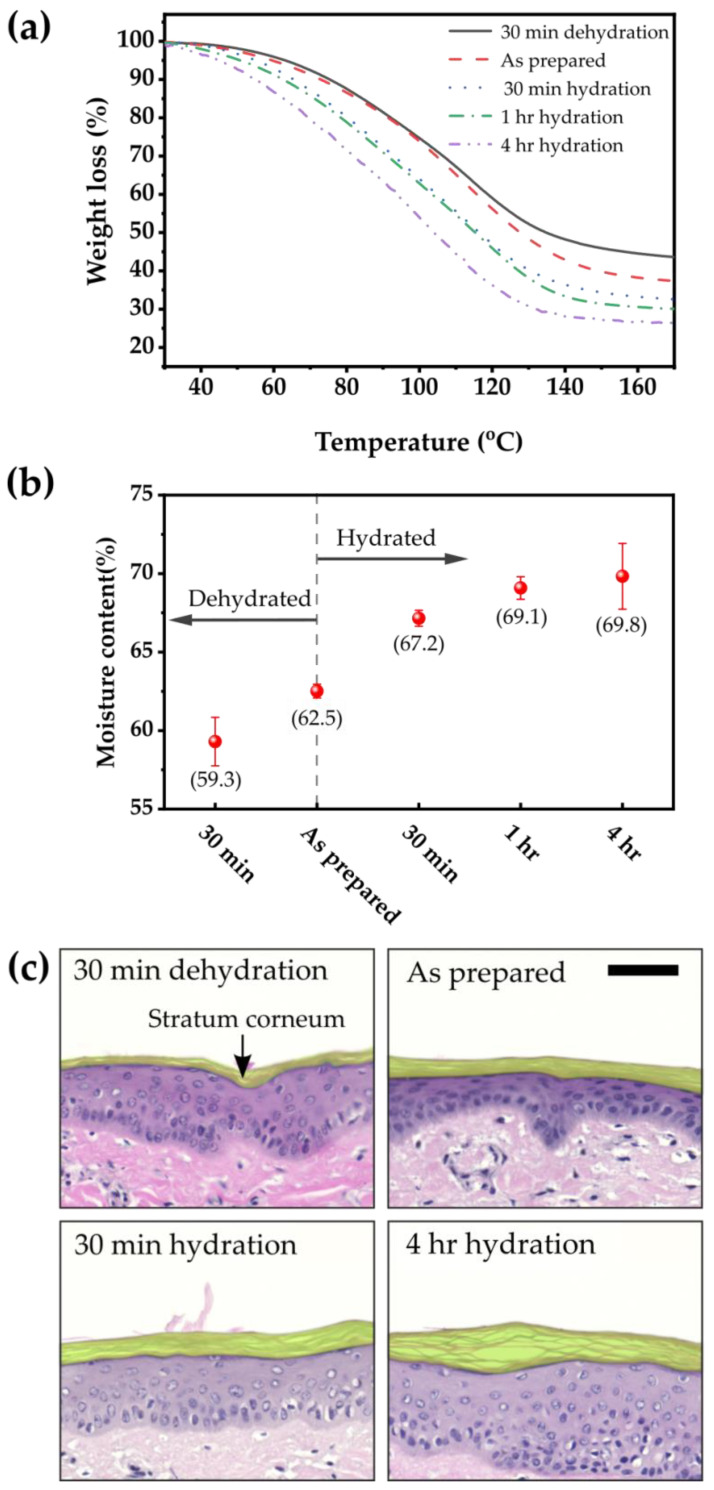
(**a**) TGA curves and (**b**) the measured moisture contents of dehydrated or hydrated porcine skin. (**c**) Histological images of porcine skin with various moisture contents. The scale bar is 50 μm.

Porcine skin was used as a model system because of its anatomical and physiological similarities with human skin [27]. To vary the hydration level of the porcine skin, it was dried for 30 min or soaked in a saline solution for 30 min, 1 h, or 4 h. Figure 3a,b show the results of TGA of the porcine skin after the hydration and dehydration processes. The “as prepared” skin sample, i.e., porcine skin as thawed, contains 62.5% moisture. When dried for 30 min, the moisture content decreases to 59.3%. As the skin becomes more hydrated in the saline solution, the moisture content increases, resulting in 69.8% of moisture content after soaking for 4 h. The histological images of the skin cross-section shown in Figure 3c also reveal that the skin becomes more hydrated and swollen with soaking time. For example, the thickness of the SC layer of the porcine skin (the outermost yellow layer) increases by swelling as the moisture content of the skin increases. When dried for 30 min, the thickness of the SC layer is about 8.3 μm; when immersed in the saline solution for 4 h, the thickness of the SC layer is about 32.8 μm, which is approximately four times thicker than the former.

### 3.3. Impedance Analysis of Hydrated Porcine Skin Using a Textile-Based Skin Hydration Sensor

**Figure 4:**
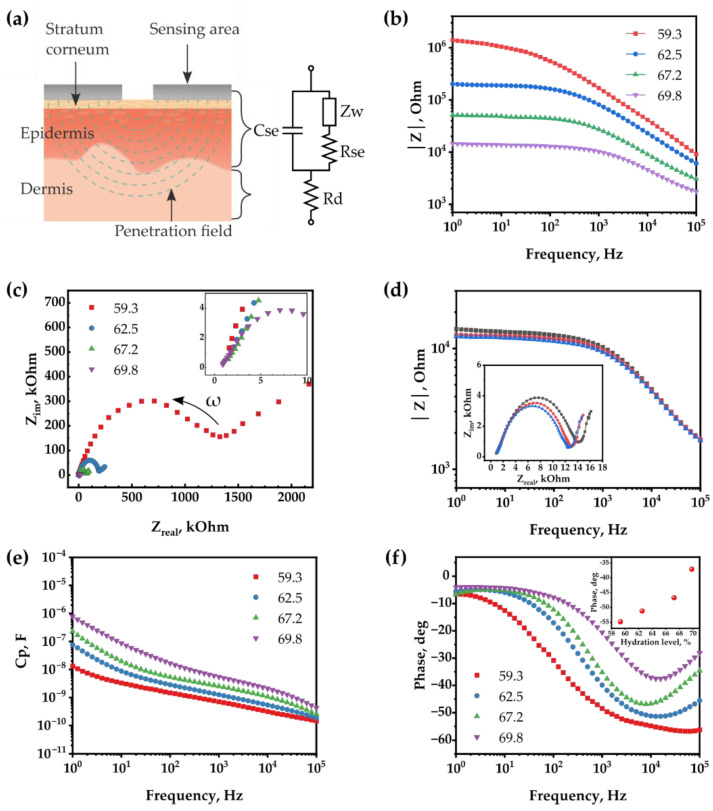
(**a**) Cross-sectional illustration of the skin–electrode system and its equivalent circuit. (**b**) Measured impedance according to the moisture content of the skin. (**c**) Nyquist plot. (**d**) Repeated measurement of total impedance and Nyquist plot (inset). (**e**) Capacitance changes and (**f**) phase values that vary with moisture content and frequency applied.

In general, the skin of mammals, including humans and porcine, consists of three major layers: SC, epidermis, and dermis. SC is the outermost layer composed of dead cells with no nucleus due to the complete differentiation of keratinocytes from the epidermis [28]. This layer has a thickness of several tens of micrometers, low moisture content, and impedance characteristics distinct from the epidermis composed of viable cells [23]. The epidermis below the SC is a hydrophilic layer in which keratinization of viable keratinocytes occurs and greatly influences skin impedance. Lastly, the deepest layer of the skin, the dermis, contains nerve components, blood vessels, sweat glands, and hair follicles. Figure 4a shows a schematic illustration of a system for measuring skin impedance using a textile-based skin hydration sensor and the corresponding equivalent circuit. In this skin–electrode system, the resistance and capacitance occurring at the interfaces of the electrodes, the SC and epidermis can be expressed by a resistor and a capacitor with C_se_ and R_se_ [29]. The Warburg impedance Z_w_ in the equivalent circuit is related to the diffusion of reactive species and ions in the skin, which is recognizable at low frequencies [30]. The dermis and underlying tissues are expressed as a pure resistor with R_d_, so the entire system is simplified to C_se_-Z_w_ and R_se_ connected in parallel and R_d_ connected in series [29].

An in-depth analysis of EIS was performed to investigate the effect of the skin’s hydration level on the impedance characteristics. First, a Bode magnitude plot of total impedance (|Z|) is depicted in Figure 4b for the porcine skin with four different moisture contents, i.e., 59.3, 62.5, 67.2, and 69.8%. The sets of skin samples correspond to 30 min dehydration, as prepared, 30 min hydration, and 4 h hydration, respectively. The skin sample of 1 h hydration was excluded as its moisture content is similar to that of the 4 h hydration skin sample. The magnitude of the impedance of the skin decreases as the moisture content of the skin increases due to the increased conductance of skin upon hydration. Thus, |Z| measured by the textile-based skin hydration sensor can be a direct indicator to monitor the hydration level of skin. For more detailed information on impedance components, a Nyquist plot is presented in Figure 4c for the skin with different hydration levels. The two x-intercepts of each semicircle represent “the bulk resistance of the skin, especially the resistance of dermis (R_d_)” at the high frequency (left side) and “R_d_ + R_se_” at low frequency (right side). Since the x-intercept for R_d_ does not change regardless of the moisture content, the dermis is not significantly affected by the skin’s hydration level. However, the diameters of the semicircles, representing R_se_, decrease as the moisture content increases. The decrease in R_se_ with increasing skin hydration level corresponds with the decrease in total impedance, as shown in Figure 4b. To confirm the stability of the sensor, the impedance of the skin with different hydration levels was measured three times for each level. Figure 4d shows the total impedance and Nyquist plot for the skin sample with 69.8% moisture content. The result shows the sensor is highly stable and reliable.

In addition to the resistance R_se_, the capacitance C_se_ of the equivalent capacitor also depends on the moisture content and AC frequency, as shown in Figure 4e. First, it is observed that C_se_ decreases as the frequency increases at all levels of skin hydration. This behavior can be explained by decreased dielectric permittivity of biological tissue with increasing frequency [31,32]. That is, the decrease in dielectric permittivity results in the decrease in capacitance [10]. In addition to the frequency, skin hydration level also influences the C_se_ values. The C_se_ values increase with the hydration level of the skin, probably due to the increase in the dielectric constant of the SC and epidermis.

Figure 4f shows the Bode phase plot, showing whether resistive or capacitive behavior is more dominant. The phase closer to 0° (90°) represents the current flows more dominantly through the resistor-like (capacitor-like) impedance element. In the plot, the phase decreases as the AC frequency increases to 10^4^ Hz. This results from the fact that the capacitive reactance of the C_se_ capacitor, which is connected in parallel with the R_se_ resistor in Figure 4a, is inversely proportional to the AC frequency. As a result, the current becomes more affected by the capacitor than the resistor at higher AC frequency, and therefore the phase approaches 90°. Noticeably, as the moisture content increases, the phase stays close to 0° for the wider AC frequency range. The plot in the inset of Figure 4f displays the phase at 10 kHz depending on the hydration level for clear demonstration of higher capacitive tendency at higher hydration level. This is because, as the skin contains more water, the R_se_, which is greatly affected by the water content, decreases. As a result, the phase shows a clear dependence on the moisture content of skin above 10^2^ Hz.

In summary, from the impedance analysis, we found that (1) R_d_ is relatively small and insensitive to the hydration level of the skin; (2) the total impedance is mainly determined by the impedance elements in SC and epidermis; and (3) the hydration level of skin can be monitored from various impedance parameters, such as R_se_, C_se_, the resulting |Z|, and phase shift.

### 3.4. Flexibility and Durability of the Textile-Based Skin Hydration Sensor

**Figure 5:**
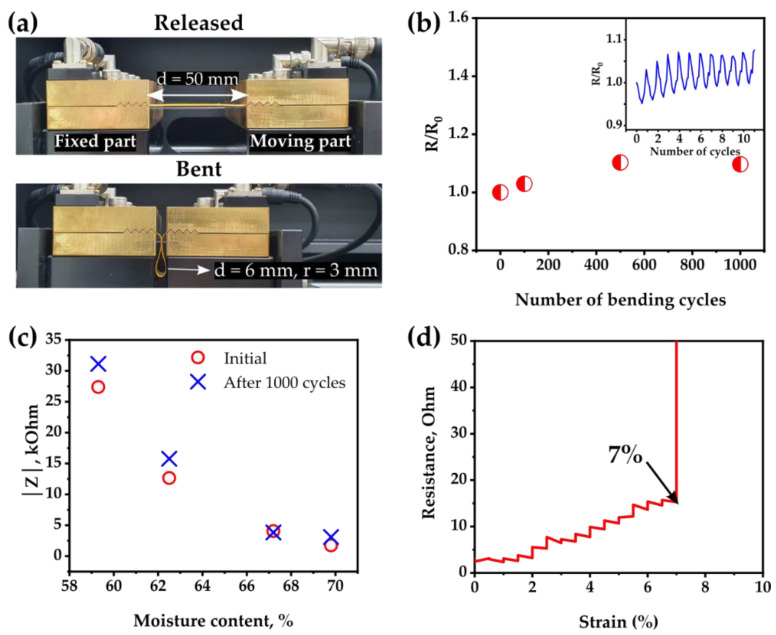
(**a**) Experimental setup of cyclic bending test for the verification of durability of the sensor. (**b**) Change in the resistance of the electrode after repetitive bending of the sensor. (**c**) Comparison of the impedance values of porcine skin with different hydration levels at 100 kHz before and after 1000 times of bending. (**d**) Change in the resistance of the electrode while applying tensile strain.

For the wearable sensor for skin, the flexibility and durability against physical deformation of the sensor are crucial for forming a conformal contact with the surface of curved body parts and reliable sensing performance. The stability of the electrical signals of the sensor was investigated under repetitive bending cycles to analyze the durability of the textile-based skin hydration sensor against physical deformation. Figure 5a shows the experimental setup where the sensing area of the sensor is placed to face upward. Figure 5b shows the change in resistance of the electrode of the sensor during 1000 bending cycles compared with the initial value. The number of cycles we selected (i.e., 100, 500 and 1000 times) are representatives to show short-, medium- and long-term changes in the resistance of electrodes. The inset shows the resistance of the electrode for the first ten cycles. The resistance slightly increases during up to 500 bending cycles and then stabilizes. After 1000 times of bending, the resistance of the electrode increases only by 9%. Figure 5c compares the |Z| values of the porcine skin samples measured by the sensor before and after 1000 bending cycles. No significant difference in the |Z| values upon the bending cycles is observed. The slight increase in the |Z| values by the bending cycles probably results from the increased resistance of the electrodes, as shown in Figure 5b. The sensor is flexible and durable enough to provide a stable electrical signal under repetitive bending cycles. Since the sensor is required to be conformally attached to the curved surfaces of skin, the electrodes also experience tensile strain. Figure 5d shows the resistance change in the electrode while applying tensile strain. The resistance drastically increases at about 7% strain. The gentle and moderate movement of the skin is not expected to degrade the performance of the sensor.

### 3.5. Demonstration of the Textile-Based Skin Hydration Sensor on Human Skin

**Figure 6:**
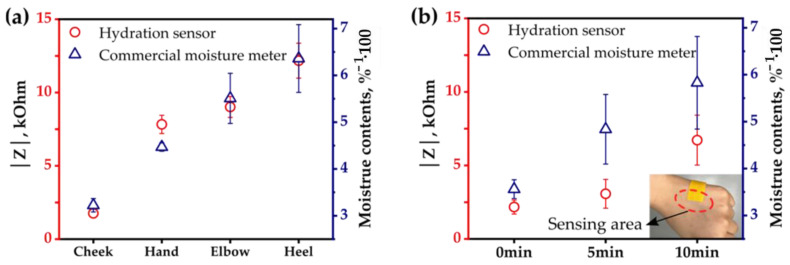
(**a**) Impedance of four different body parts. (**b**) Impedance of a hand after washing. The impedance is measured with the textile-based skin hydration sensor at a fixed frequency of 100 kHz and is plotted with the readings from a commercial moisture meter.

To demonstrate the usability of the textile-based sensor for human skin, two different sets of experiments were conducted: (1) measurement of the impedance on various parts of the human body and (2) time-dependent monitoring of impedance on the skin of hands. We measured the impedance of skin on four different body parts (cheek, hand, elbow, and heel) by using the textile-based hydration sensor (Figure 6a). The hydration level of each body part varies depending on the degree of callus formation and skin care habits [33]. For example, elbows and heels that experience more frequent frictions and thick calluses are expected to have lower hydration levels than cheeks. As expected, the cheeks show the lowest impedance, ~2 kΩ, and the heels show the highest impedance, ~12 kΩ. The total impedance |Z| values show a larger standard deviation for body parts with lower skin moisture content. It seems that the SC of skin with the less moisture content (i.e., rough and cracked skin) affects the adhesion of the sensor. The |Z| values are compared to the reciprocal of the moisture contents measured by the commercial sensor because the |Z| values are inversely dependent on the hydration level. The impedance values measured by the textile-based sensor coincide with the reciprocal values of the moisture contents readings from the commercial sensor. We also monitored the hydration level of the hand with time by measuring the skin impedance with the textile-based hydration sensor. The impedance on the back of the hand as a function of elapsed time after washing is compared with the reciprocal of readings from the commercial sensor shown in Figure 6b. The impedance gradually increases with time because the skin’s hydration level decreases over time after hand washing. The commercial sensor shows a similar trend to the textile-based sensor. Notably, the textile-based hydration sensor shows more reliable results with fewer standard deviations than the commercial sensor during repeated measurements. The better measurement reproducibility by the textile-based skin hydration sensor could be due to the stable conformal contact with minimal pressure applied to the skin. Thus, together with their time-dependent changes, the textile-based sensor could effectively detect the different hydration levels of human skin on various body parts.

## 4. Conclusions

In this study, we present a textile-based wearable sensor and its sensing mechanism of hydration level inside the skin based on the impedance analysis. The textile-based sensor was fabricated by printing the conducting patterns with well-designed dimensions on a cotton substrate. The porcine skin with different hydration levels was prepared as a model system of the skin. Impedance characteristics of the porcine skin samples were investigated using textile-based sensors. The EIS shows that (1) the total impedance of skin decreases as its hydration level increases, and (2) the impedance of the SC and epidermis layers are more affected by the moisture contents in the skin than the dermis layer. The durability of the sensor against physical deformation was validated through a cyclic bending test. Lastly, the textile-based sensor demonstrates consistent sensing of hydration levels in various parts of human skin. The measurements are reliable due to the conformal contact with skin, and the resulting data are well-matched with the readings from the commercial sensor. The fabrication process of the textile-based sensor is simple and scalable, and its production cost could be very low. The textile-based skin hydration sensor can also be incorporated into conventional textile products for biomedical and health care applications. Further investigation on the stability of the sensor is now underway by monitoring the morphology change in all components, especially the electrodes, during long-time operation. Moreover, the electrode design could be more intricate to enhance sensing performance or to be adjusted to the user’s skin conditions.
